# Results of NC-6300 (Nanoparticle Epirubicin) in an Expansion Cohort of Patients with Angiosarcoma

**DOI:** 10.1093/oncolo/oyac155

**Published:** 2022-08-03

**Authors:** Richard F Riedel, Victoria Chua, Ania Moradkhani, Natalie Krkyan, Amir Ahari, Atsushi Osada, Sant P Chawla

**Affiliations:** Duke Cancer Institute, Duke University Medical Center, Durham, NC, USA; Sarcoma Oncology Research Center, Santa Monica, CA, USA; Sarcoma Oncology Research Center, Santa Monica, CA, USA; Sarcoma Oncology Research Center, Santa Monica, CA, USA; Sarcoma Oncology Research Center, Santa Monica, CA, USA; NanoCarrier Co., Ltd., Tokyo, Japan; Sarcoma Oncology Research Center, Santa Monica, CA, USA

**Keywords:** nanoparticle, epirubicin, angiosarcoma, metastatic, soft tissue sarcoma, unresectable, NC-6300

## Abstract

**Background:**

NC-6300 is a novel epirubicin (EPI) drug conjugated polymeric micelle developed using cutting-edge micellar nanoparticle technology. The nanoparticle epirubicin conjugates EPI to a polymer via a pH-sensitive linker which enables the selective EPI release into tumor. Tumor activity was observed in a monotherapy phase Ib trial, where two of two patients with angiosarcoma achieved a partial response. To further explore the activity of NC-6300 in angiosarcoma, an expansion cohort was undertaken.

**Methods:**

Ten patients with angiosarcoma were enrolled in the expansion cohort. Patients were dosed using the recommended dose of 150 mg/m^2^ intravenously (IV) once every 3 weeks. The primary endpoint was progression-free survival.

**Results:**

The most common adverse events (AEs) of any grade, regardless of the causal relationship with NC-6300, were neutropenia (90%), fatigue, and thrombocytopenia (60% each) and nausea (50%). The most common grades 3 and 4 AEs were neutropenia (80%), thrombocytopenia (40%), and anemia and leukopenia (20% each). The median progression-free survival (mPFS) for all subjects was 5.4 months. The mPFS was 3.8 months in subjects with prior anthracycline treatment and 8.2 months in subjects without prior anthracycline treatment.

**Conclusion:**

NC-6300 was well tolerated, showing promising activity in angiosarcoma patients without prior anthracycline treatment. NC-6300 warrants further investigation (ClinicalTrials.gov Identifier: NCT03168061).

## Teaser

To explore the activity of NC-6300 (nanoparticle epirubicin) in angiosarcoma, an expansion cohort was undertaken. The clinical trial results are reported here.

**Lessons Learned**

- In this small expansion cohort, NC-6300 at the recommended dose of 150 mg/m^2^ IV every 3 weeks, was well tolerated and showed promising anti-tumor activity in angiosarcoma patients without prior anthracycline treatment.

## Discussion

Angiosarcomas are a subtype of soft-tissue sarcoma that are aggressive, malignant endothelial-cell tumors of vascular or lymphatic origin. Treatment for patients is challenging in many cases and the prognosis is poor. Anthracyclines are widely used in the treatment of many sarcomas, including angiosarcomas, but many patients suffer from rate-limiting side effects. Therefore, a safer treatment remains an area of unmet medical need. EPI, a stereoisomer of doxorubicin, was developed to circumvent the cardiotoxicity associated with the parent compound. Pegylated liposomes and other nanoparticle formulations have been developed to reduce the cardiotoxicity of conventional doxorubicin and other anthracycline derivatives. Although liposomal doxorubicin was designed to alleviate the cardiac toxicity associated with conventional doxorubicin, unique toxicities were reported. NC-6300 was developed to encapsulate EPI into the micellar nanoparticle to selectively deliver the drug into the acidic environment of the endosomal or lysosomal compartments of the target tumor cell.

**Figure 1.**
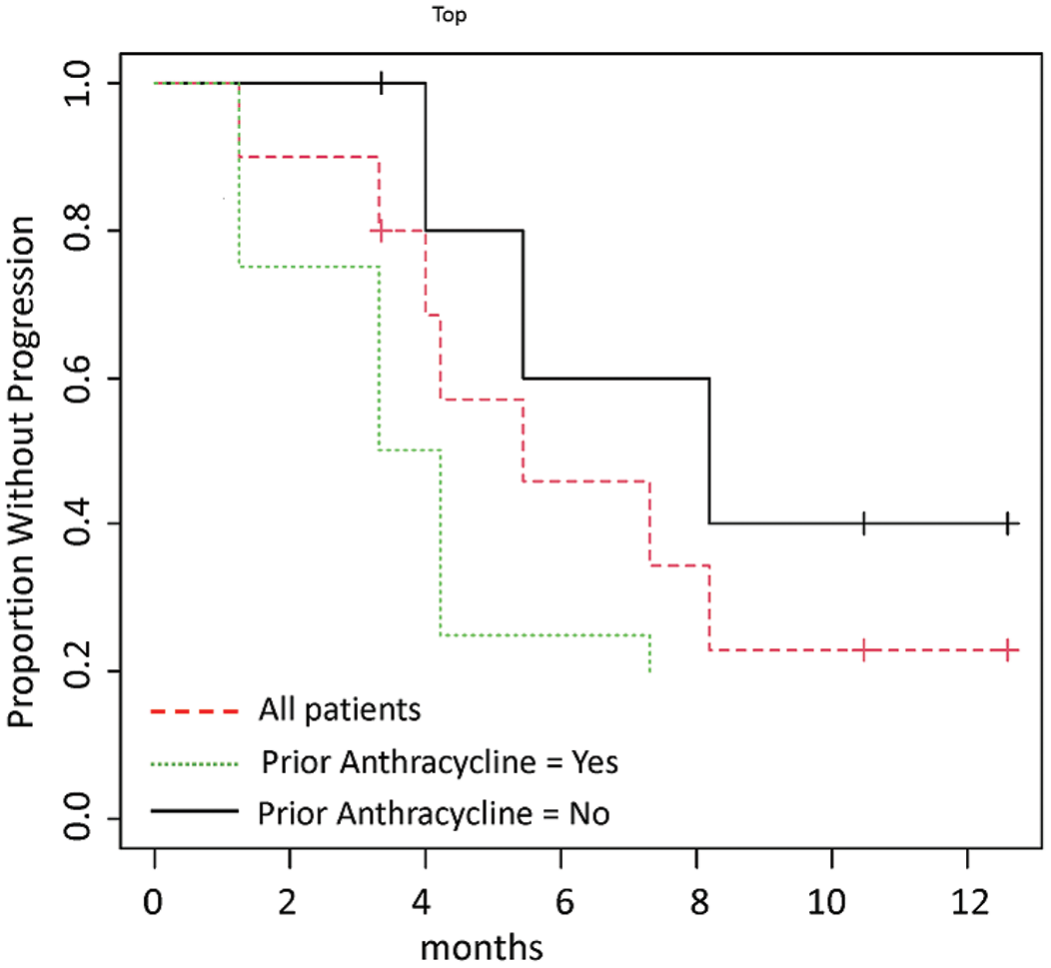
Progression-free survival (*N* = 10). The overall mPFS for all subjects included in the trial was 5.4 months. The mPFS was 3.8 months in subjects with prior anthracycline treatment and 8.2 months for subjects without prior anthracycline treatment.

In the phase Ib trial of NC-6300 in solid tumors and soft-tissue sarcomas, there were 2 patients with angiosarcoma who experienced a partial response by investigator assessment. Originally, a comparative phase II accruing 150 patients in total was planned to investigate NC-6300 + olaratumab vs olaratumab alone. However, the study protocol was amended to pursue an expansion cohort, using NC-6300 alone, due to market withdrawal of olaratumab. The expansion cohort was an open-label trial that enrolled 10 patients designed to further investigate the activity of NC-6300 in angiosarcoma. The primary endpoint was progression-free survival (PFS). Patients were treated with NC-6300 at 150 mg/m^2^ IV once every three weeks. The baseline characteristics of the patients are listed in Table 1. The results of our trial demonstrated the tolerability and the safety of NC-6300, with the most common adverse events ≥30% in all grades being neutropenia (90%), thrombocytopenia (60%), fatigue (60%), and nausea (50%). Only neutropenia (80%) and thrombocytopenia (40%) had occurrences in grade 3 or 4 ≥30%. The overall mPFS by investigator assessment was 5.4 months. The mPFS was 3.8 months in subjects with prior anthracycline treatment and 8.2 months for subjects without prior anthracycline treatment as shown in Figure 1. Partial response was observed in 3 patients without prior anthracycline treatment, yielding an objective response rate of 30% (3/10).

**Table 1.** Patient baseline characteristics (*N* = 10).

Characteristics	No. of patients	%
No. of patients enrolled	10	100
Age, years, median (range)	63.5 (26-76)	
Sex		
Male	7	70
Female	3	30
ECOG PS		
0	1	10
1	9	90
Angiosarcoma variants		
Cutaneous	2	20
Non-cutaneous	8	80
Previous systemic chemotherapy		
No	3	30
Yes	70	70
Median (range)	1 (0-5)	
Previous anthracycline chemotherapy		
No	6	60
Yes	4	40
Agents used in prior regimens		
Doxorubicin	4	40
Gemcitabine	4	40
Paclitaxel	4	40
Docetaxel	3	30
Pazopanib	2	20
Ifosfamide	1	10
Liposomal doxorubicin	1	10

In summary, NC-6300 was safe and tolerable in this expansion cohort, and had promising efficacy especially in angiosarcoma patients without prior anthracycline treatment. Further investigation is warranted.

## Trial Information

**Table:** 

Disease	Angiosarcoma
Stage of disease/treatment	Recurrent or metastatic setting
Prior therapy	Chemotherapy-naïve or no more than two previous systemic therapies for recurrent or metastatic setting
Type of study	Interventional
Primary endpoint	Median PFS
Secondary endpoints	Safety profile (the incidence, severity, seriousness, and relatedness to study drug of TEAEs, laboratory changes or LVEF)
	ORR, DCR (ORR + stable disease) at 4 months
	DOR
	Median OS
Investigator’s analysis	Active and should be pursued further

## Drug Information

**Table:** 

Generic/working name	NC-6300
Company name	NanoCarrier Co., Ltd
Drug type	Chemotherapy
Drug class	Anthracycline
Dose	150
Unit	mg/m^2^
Route	Intravenous infusion
Schedule of administration	Every 3 weeks

## Patient Characteristics

**Table:** 

Number of patients, male	7
Number of patients, female	3
Age: median (range)	63.5 (26-76) years
Number of prior systemic therapies: median (range)	1 (0-5)
Performance status: ECOG	0: 1
	1: 9
	2: 0
	3: 0
	4: 0
Cancer types or histologic subtypes	Angiosarcoma cutaneous, 2; Angiosarcoma non-cutaneous, 8

## Primary Assessment Method

**Table:** 

Title	Imaging (CT or MRI)
Number of patients screened	10
Number of patients enrolled	10
Number of patients evaluable for toxicity	10
Number of patients evaluated for efficacy	10
Evaluation method	RECIST 1.1
Response assessment, CR	0 (0%)
Response assessment, PR	3 (30%)
Response assessment, SD	6 (60%)
Response assessment, PD	1 (10%)
Median duration assessments	Every 6 weeks

## Assessment, Analysis, and Discussion

**Table:** 

Completion	Study completed
Investigator’s assessment	Active and should be pursued further

Angiosarcomas are a subtype of soft-tissue sarcoma that are aggressive, malignant endothelial-cell tumors of vascular or lymphatic origin. Anthracyclines, such as doxorubicin, are widely used in the treatment of many tumors, including angiosarcomas, but many patients suffer from significant treatment-related side effects. Treatment for patients is challenging in many cases and the prognosis is poor. As a result, there remains an unmet need for a safer anthracycline agent. Epirubicin, a novel stereoisomer of doxorubicin, was developed to circumvent the cardiotoxicity associated with the parent compound. Pegylated liposomes and other nanoparticle formulations have been developed to reduce the cardiotoxicity of conventional doxorubicin and other anthracycline derivatives. Although liposomal doxorubicin was designed to alleviate the cardiac toxicity associated with conventional doxorubicin, some unique toxicities were observed, including acute infusion-related reaction and Hand-Foot Syndrome, which were not common with conventional doxorubicin. NC-6300 was developed to encapsulate epirubicin into the micellar nanoparticle to selectively deliver the drug into the acidic environment of the endosomal or lysosomal compartments of the target tumor cell. NC-6300 was also designed to stay in the blood circulation for a long period of time and accumulate in the tumor based on the enhanced permeability and retention effects. Superior anti-tumor activity of NC-6300 was expected due to efficient drug release in the tumor.

The phase Ib does-escalation trial of NC-6300 monotherapy (NCT 03168061) enrolled 29 subjects: 17 with soft-tissue sarcoma, 1 with osteosarcoma, and 11 with other solid tumors. Among those patients, there were 2 patients with angiosarcoma who experienced a partial response by investigator assessment. Originally, a comparative phase II study, accruing 150 patients in total, was planned to investigate NC-6300 + olaratumab vs olaratumab alone. However, the study protocol was amended to pursue an expansion cohort, exploring NC-6300 alone in angiosarcoma, due to market withdrawal of olaratumab. The expansion cohort of angiosarcoma was an open-label, non-randomized trial that enrolled 10 patients designed to further investigate the activity of NC-6300. The primary endpoint of the expansion cohort was progression-free survival (PFS). Patient visits took place at screening; on days 1, 8, and 15 of every cycle; and end of treatment. AEs were graded using the NCI Common Terminology Criteria for Adverse Events Version 4.03 (CTCAE v4.03). Patients were treated with NC-6300 at 150 mg/m^2^ IV once every 3 weeks. The baseline characteristics of the patients are listed in Table 1. All subjects enrolled had a histologically or cytologically confirmed diagnosis of advanced solid tumor, including soft-tissue sarcoma, that had relapsed or was refractory to standard therapy, and an Eastern Cooperative Oncology Group (ECOG) performance status, defined as 0 or 1. Overall, 70% of subjects were men and 30% were women. Median age was 63.5 years (range: 26–76 years). Eighty percent of the enrolled subjects had non-cutaneous angiosarcomas and 20% had cutaneous angiosarcomas. Four subjects (40%) had received prior anthracycline chemotherapy and six subjects (60%) had not received prior anthracycline chemotherapy; 3 subjects had no previous systemic chemotherapy treatment prior to enrolling in the study; 7 subjects had previous systemic chemotherapy treatment. The agents used in the prior treatment included: taxane (70%), doxorubicin (40%), gemcitabine (40%), pazopanib (20%), ifosfamide (10%), and liposomal doxorubicin (10%).

Although immunotherapy has significantly changed the standard of care for many advanced malignancies, neither all patients nor all disease states benefit. As a result, chemotherapy remains an important treatment modality for many individuals with advanced disease. Anthracyclines, including doxorubicin, are some of the most commonly used chemotherapeutic agents, but are limited by hematologic and cardiac toxicities. As a result, novel anthracyclines, such as epirubicin, were developed.

While doxorubicin-based chemotherapy remains a treatment of choice in metastatic soft tissue sarcomas, paclitaxel seems to be an effective treatment, specifically in angosarcoma. In a multicenter phase II trial to assess the efficacy and toxicity of weekly paclitaxel in patients with metastatic or unresectable angiosarcomas, a median time to progression of 4 months was observed.^1^ Progression-free survival was similar in patients pre-treated with chemotherapy and in chemotherapy-naïve patients.^1^

Our expansion trial demonstrated the tolerability and safety of NC-6300 with the most common adverse events ≥30% in all grades being neutropenia (90%), thrombocytopenia (60%), fatigue (60%), and nausea (50%). The most common grades 3 and 4 AEs were neutropenia (80%), thrombocytopenia (40%), and anemia and leukopenia (20% each). Adverse events regardless of the causal relationship with NC-6300 are listed in Table 2. These toxicities are commonly observed with conventional anthracycline treatment.^2^

**Table 2.** NC-6300 safety results (*N* = 10).

Adverse events*	Grade							
	1 and 2		3		4		All	
	No.	%	No.	%	No.	%	No.	%
Neutropenia	1	10	1	10	7	70	9	90
Fatigue	6	60	0	0	0	0	6	60
Thrombocytopenia	2	20	2	20	2	20	6	60
Nausea	5	50	0	0	0	0	5	50
Anemia	2	20	2	20	0	0	4	40
Decreased appetite	4	40	0	0	0	0	4	40
Stomatitis	4	40	0	0	0	0	4	40
Constipation	3	30	0	0	0	0	3	30
Cough	3	30	0	0	0	0	3	30
Diarrhea	3	30	0	0	0	0	3	30
Leukopenia	1	10	1	10	1	10	3	30
Pyrexia	3	30	0	0	0	0	3	30
Vomiting	3	30	0	0	0	0	3	30
Headache	2	20	0	0	0	0	2	20
Hypomagnesaemia	2	20	0	0	0	0	2	20
Insomnia	2	20	0	0	0	0	2	20

*Adverse events regardless of causal relationship with NC-6300

**Figure 2.**
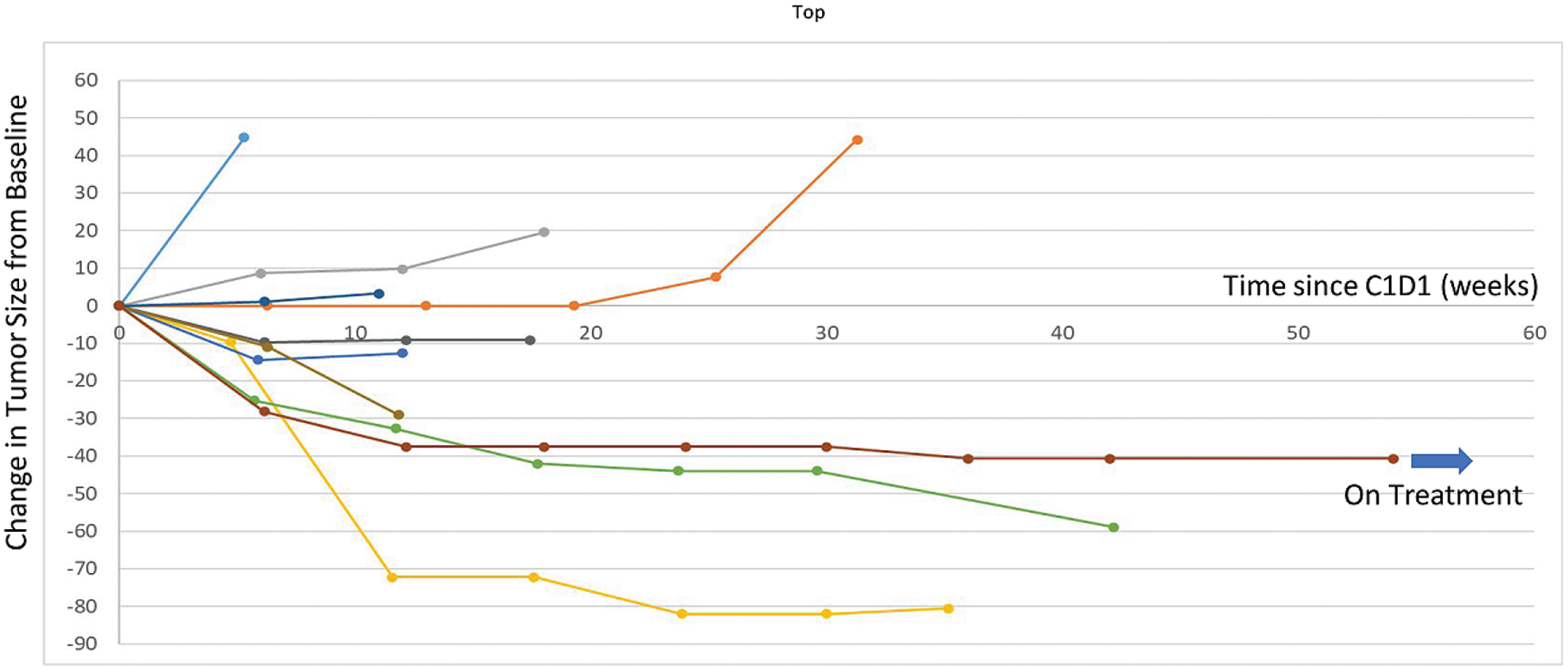
Spider plot of maximal change in tumor size in angiosarcoma population (*N* = 10). Partial response was observed in 3 patients without prior anthracycline treatment, yielding an objective response rate (ORR) of 30%.

The Kaplan-Meier method was used to calculate the median progression-free survival. The overall mPFS by investigator assessment for all subjects included in the trial was 5.4 months, similar to what was achieved, in prior published studies, with anthracycline (4.9 months) and paclitaxel regimens (4 months) for patients with metastatic or unresectable angiosarcomas.^1,3,4^ In subjects with prior anthracycline treatment, the mPFS in the study presented here was 3.8 months, similar to the expected mPFS for treatment of angiosarcoma (3.5 months, 3.7 months, and 2.7 months in first-, second-, and third-line setting).^5^ In contrast, in subjects without prior anthracycline treatment, the mPFS was 8.2 months, as shown in Figure 1. All subjects were evaluated for a radiographic assessment of tumor response using RECIST version 1.1. Partial response was observed in 3 patients without prior anthracycline treatment, yielding an objective response rate of 30% (3/10) (Figure 2). Even in this small sample size, the ORR is similar to what has been previously reported for first-line paclitaxel (23.7%).^1^

NC-6300 at the recommended dose of 150 mg/m^2^ IV every 3 weeks was well tolerated and showed promising anti-tumor activity in patients without prior anthracycline exposure in this expansion cohort. Further investigation of the activity of NC-6300 is warranted.

## Data Availability

This study is still in progress. Once it completes, NanoCarrier will make the data public by entering into ClinicalTrials gov. website.
